# Predictions of Crack Growth Rates, R-Ratio and Overload Effects Based on Smooth Specimen LCF Data and the Moving Plastic Stress Field Ahead of the Crack Tip

**DOI:** 10.3390/ma19112411

**Published:** 2026-06-05

**Authors:** Steve Williams, Mark Whittaker, Mark Hardy

**Affiliations:** 1 Institute of Structural Materials, Swansea University, Fabian Way, Swansea SA1 8EN, UK; m.t.whittaker@swansea.ac.uk; 2 Rolls-Royce plc, P.O. Box 31, Moor Lane, Derby DE22 8BJ, UK; mark.hardy@rolls-royce.com

**Keywords:** crack growth, LCF, strip yield models, plasticity, R-ratio effects, overloads

## Abstract

The use of the stress intensity factor K to characterize the severity of crack tip stress fields is widespread throughout engineering. The relationship between K and the crack growth rate is then usually represented empirically by a straight line Paris law relationship on logarithmic axes. This study develops an analytical relationship between the two by linking crack growth to the accumulation of fatigue damage ahead of the moving crack tip. A stress-based fatigue model was used, with inputs from plastic 2D plane stress FE analyses representing an edge crack by a sharp semi-circular notch. Stress–distance profiles ahead of the crack tip were extracted at the maximum and minimum points of a range of fatigue loading cycles. These were then used with data from smooth specimen LCF tests to predict the build-up of fatigue damage at regularly spaced locations ahead of the crack tip and hence crack growth rates. Full da/dN–ΔK curves were generated for the nickel-based superalloy RR1000 at 20 °C with loading R-ratios of 0, −1 and 0.5. The R = 0 and R = −1 crack growth rate predictions agreed well with experimental data, as did the steeper growth rate slope calculated at R = 0.5. The method was then extended to predict overload behaviour.

## 1. Introduction

As part of a wider programme to investigate crack growth phenomena such as closure, R-ratio (min/max load) effects, overload retardation and high-temperature tunnelling, non-linear FE analyses using the ABAQUS 2021 code have been run on a range of 2D and 3D models containing sharp notches representing cracks.

This paper takes the predicted maximum and minimum plastic stress fields from 2D plane stress analyses on geometry representing an edge crack together with data from smooth specimen LCF tests to predict the build-up of fatigue damage at evenly spaced locations ahead of the crack tip (notch root) as it moves towards them. When the fatigue damage at a location reaches unity, the material there is considered to have failed, and hence the crack has reached that position. Knowing the number of fatigue cycles required to fail the material at successive calculation locations then allows crack growth rates to be calculated.

The complexity of the stress fields and material behaviour close to the crack tip is extremely high and can prevent any progress being made if a model attempts to include everything in its formulation. Accordingly, this work initially aimed to demonstrate the concept of the calculation method by using a number of appropriate simplifying assumptions. Additional complexity could then be included as necessary in aspects such as the finite element, cyclic plasticity and fatigue damage models depending on the trends in the results obtained. Overall, the aim of developing this fatigue analogy model was to provide further insight on a number of aspects of crack growth behaviour that are usually represented empirically such as the overall shape of the da/dN–ΔK curve, R-ratio effects and propagation rates following an overload cycle.

The material studied was the γ’ strengthened nickel-based superalloy Fine Grain (FG) RR1000 at 20 °C. RR1000 is used for aero engine high-pressure compressor and turbine discs, and different heat treatments are used to produce two variants of the material for specific applications. Fine Grain RR1000 has high tensile strength, allowing certification overspeed regulations to be met with lightweight disc designs. There is also a Coarse Grain (CG) version which is optimised for high-temperature crack growth resistance.

Many of the analytical methods for understanding crack tip stress fields and phenomena such as closure and overload behaviour are based on the strip yield model originally developed by Newman [1]. This represents the crack and the plastic zone ahead of the crack tip by a series of elements that support a 1D stress field in the loading direction and have finite widths. The material’s deformation is usually assumed to be solely due to plasticity, i.e., its behaviour is rigid-perfectly plastic. Creep deformation can also be included in high-temperature analyses.

Predictions of crack closure behaviour from strip yield models have been shown to line up well with experimental data in papers by many authors including Wang and Blom [2], Vidler, Kotousov and Ng [3], Rose and Wang [4] and Beretta and Carboni [5]. Measured plane stress closure loads on steel and aluminium sheet test pieces from these programmes are often high, around 40% of the maximum applied load. Work by Paluskiewicz et al. [6] on In718 alloy, however, which is a similar type of material to FG RR1000, showed no closure at a loading R-ratio (min/max load) of 0.1.

The end point of strip yield modelling work and similar studies using FE models of the crack tip region is usually to determine the effective stress intensity factor range ΔK_eff_, which represents the stress intensity range over which the crack is open. Its relationship with the maximum stress intensity in the loading cycle K_max_ and the stress intensity at which the crack opens K_op_ is shown in Equation (1).

(1) $${∆K}_{eff}=K_{max}-K_{op}$$

If the relationship between ΔK_eff_ and experimental crack growth rates is established for one R-ratio, the strip yield models then allow growth rate predictions to be made for other loading conditions.

Studies by Chen, Weiss and Stickler [7], Castro, Meggiolaro and Miranda [8], Vasudevan, Sadananda and Holtz [9] and Fleck [10] have all, however, provided examples where Δ*K_eff_* has not been able to represent aspects of crack growth behaviour correctly.

As an alternative, therefore, methods that represent crack growth as a fatigue process and use stress and/or strain-related parameters rather than stress intensities as the basis for their predictions of crack growth rate were investigated. Examples of these Critical Damage Models based on a strip yield framework have been published by Noroozi, Glinka and Lambert [11] and Ferreira, Pinho de Castro and Meggiolaro [12].

**Figure 1:**
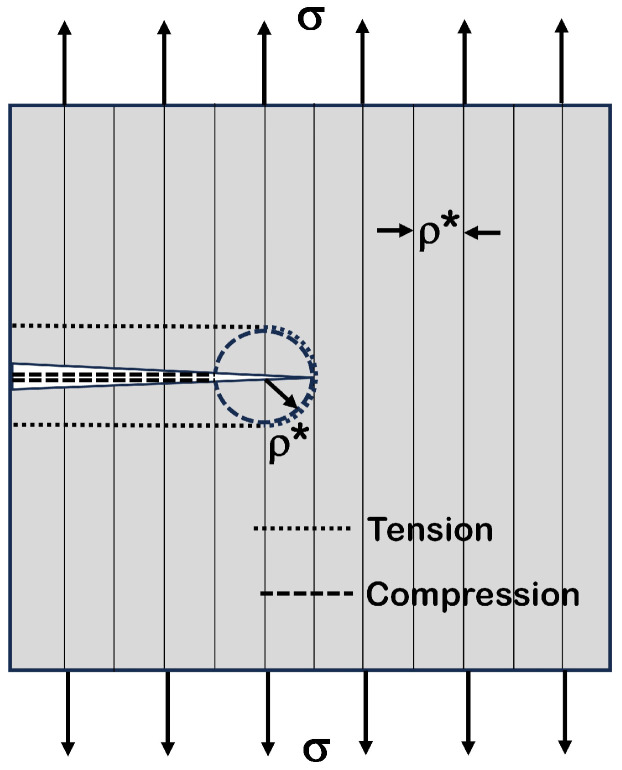
Schematic illustration of the model used by Noroozi, Glinka and Lambert [11] to predict crack tip stresses.

The basis of Noroozi’s model is shown schematically in Figure 1 below. As with the work described in this paper, the crack tip is represented as a sharp semi-circular notch. The notch radius is ρ*, and the fatigue damage is assessed using averaged loading conditions in material strips of this width ahead of the notch. The Creager–Paris [13] solution is used to determine the mean tensile elastic stresses in each strip ahead of the crack tip, and the compressive elastic stress fields are determined by treating the crack tip as a circular hole. The Neuber [14] construction and a Ramberg–Osgood [15] stress–strain curve are then used to calculate the plastic Smith–Watson–Topper [16] parameter and hence fatigue damage and crack growth rates. The dimension ρ* is used as a fitting parameter to line the predictions up with the material’s threshold stress intensity value, which is required as an input to the model.

Ferreira, Pinho de Castro and Meggiolaro [12] used both the Smith–Watson–Topper and Coffin–Manson [17] equations to calculate fatigue damage, concluding that the latter gave a better match to experimental crack growth rates. They also linked the crack growth threshold to the fatigue endurance limit and the fracture toughness to a critical level of plastic strain.

Reasonable crack growth rate predictions were made for a wide range of loading R-ratios in both papers, but it was considered that using similar crack geometry and plastic finite element analyses rather than strip yield methods to calculate the input stresses could provide additional insights on the material behaviour.

## 2. Materials, Methods, Results and Discussion

The modelling work required to predict crack growth rates under different loading regimes based on fatigue damage accumulation ahead of the crack tip was done in four distinct phases, each of which is discussed separately in this section.

Firstly, a finite element modelling framework was established that was shown to calculate geometry correction factors for edge cracks (and hence elastic stress fields) that were consistent with previously published solutions. Plastic FE analyses were then run for a wide range of K_max_ values at R = 0 to generate maximum and minimum stress–distance profiles that were used as inputs to the fatigue damage calculations.

Next, a spreadsheet-based method was developed to allow the fatigue damage accumulation at a large number of discrete material locations ahead of the crack tip to be calculated as it moves through the structure. The fatigue curve used for the damage calculations was fitted to the results of load and strain-controlled LCF tests run on plain specimens at R-ratios of 0 and −1. The method was then used to predict R = 0 crack growth rates at a wide range of K_max_ values and hence the overall shape of the da/dN–ΔK curve.

After running additional FE analyses, the method was extended to predict crack growth rates at R-ratios of −1 and 0.5.

Finally, the spreadsheet method was improved to use a finer spatial grid for the calculations and to implement the calculation process as a macro. This allowed crack growth rates following single cycle overloads to be predicted.

In each of the four following sections the calculation method is first described, followed by a comparison of the modelled results with experimental data and a discussion on the findings.

### 2.1. Finite Element Modelling to Generate Edge Crack Geometry Correction Factors and Calculate Plastic Stress–Distance Profiles

**Figure 2:**
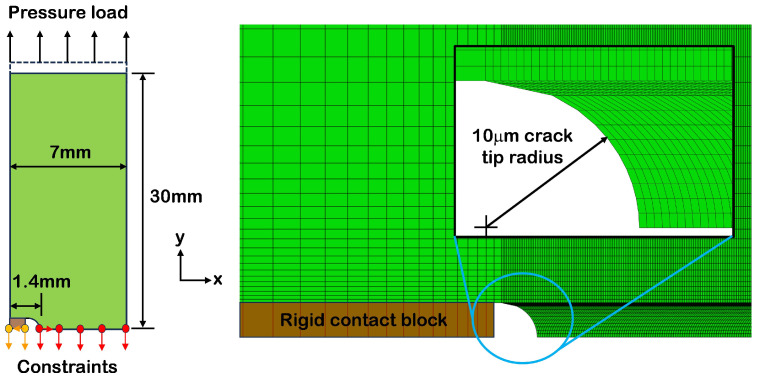
Edge crack 2D plane stress finite element model—mesh geometry and loading details.

The model used for the finite element analyses is shown in Figure 2. A 1.4 mm long notch with a 10 µm end radius is contained within a rectangular section 7 mm wide and 30 mm long. The crack length and section geometry were chosen to match an experimental condition in the overall research programme, which used corner crack test pieces with a 7 mm wide test section and threaded ends. Advantage was taken of the geometry’s symmetry to model only the top half of the crack and surrounding material. Four-noded CPS4 plane stress elements were used with linear geometry.

It was considered appropriate to use a plane stress FE model even though verification of the method uses data from corner crack tests because of the uncertain role played by the multiaxial crack tip stress field in propagating the crack. Zhang and Doré [18] and Tong [19] compared experimental data on steels generated using corner crack and single-edge notch tension and bending specimens (with 3D and 2D plane stress fields, respectively) and concluded that there was no significant influence of the different geometries on the crack growth rates.

In the event of poor crack growth rate predictions being obtained using results from plane stress FE models, however, the strategy was to re-run the analyses using the same meshes but 2D plane strain elements. Relating these results to the crack growth rates would then require shifting to a multiaxial fatigue damage model such as used by Kujawski [20].

The FE model also contains a row of fully rigid elements which runs from the crack mouth to just short of the run-out of the notch radius. The tops of these elements contact the crack face in such a way that they separate under tension but can transmit compressive stresses in the loading (y) direction if the crack is predicted to close. All the nodes on the bottom edge of the cracked geometry and contact block are fixed in the y direction, and the nodes at the notch root and the bottom right-hand corner of the contact block are also pinned in the x direction.

A uniform pressure load is applied to the top of the model, and the nodal displacements there in the loading direction are all set to be equal using the ABAQUS *EQUATION keyword. This is not strictly necessary but represents the remote boundary conditions for a threaded test piece more appropriately than allowing the nodes there to deform freely.

The geometry was generated using a macro in Microsoft Excel, and a variety of node/element patterns close to the notch root were used in the full programme. The version used for this study contains multiple layers of elements ahead of the crack tip 0.5 µm wide that, in other work, were switched off progressively as the load was cycled to simulate crack growth whilst maintaining the original crack tip radius. Finally, five rings of elements around the arc of the notch root were specified by the *CONTOUR INTEGRAL keyword to allow J-integral values to be calculated from the analyses.

**Figure 3:**
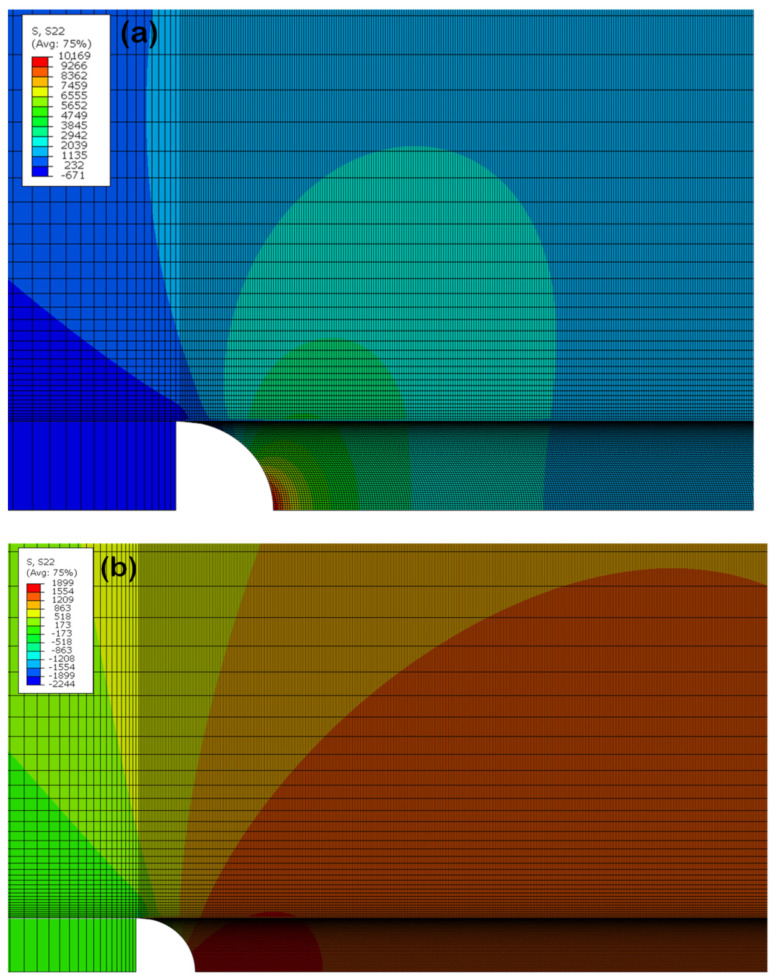
Loading direction stress contours from (**a**) elastic and (**b**) plastic FE analyses: FG RR1000 at 20 °C and K_max_ = 25.8 MPa√m.

Example stress contours in the loading direction are shown in Figure 3 from elastic (3a) and plastic (3b) FE analyses. Initial analyses had kinks in the contours around the shallow angled elements at the top of the notch radius which were largely resolved by splitting each of them horizontally into five smaller elements. This detail can be seen in the magnified region of Figure 2.

**Figure 4:**
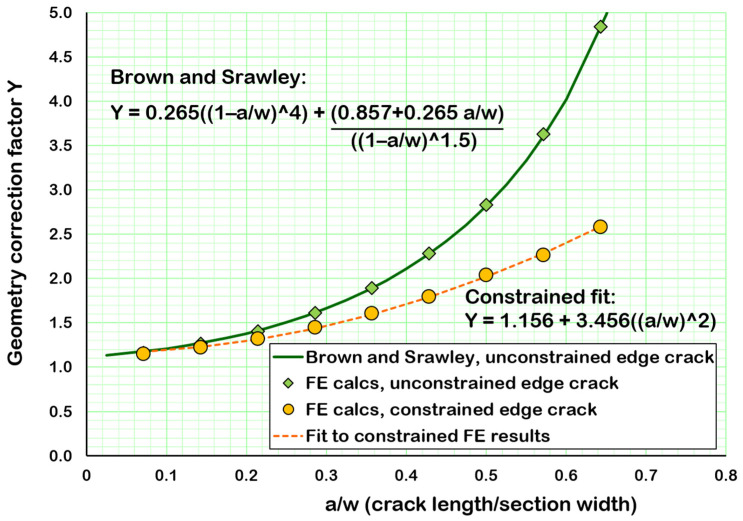
Comparison of geometry correction factors from the FE model with those from the Brown and Srawley equation.

The suitability of the sharp notch for representing the crack tip stress field was then established. Geometry correction factors Y for nine different crack lengths were calculated from the ABAQUS J-integral output for models run with no constraints on the top edge. These were compared with the standard solution for edge cracks developed by Brown and Srawley [21] as shown in Figure 4. In the figure, the data points are from the FE analyses, and the curves are generated from the geometry correction factor polynomials. The FE results are within 0.5% of the Brown and Srawley values throughout. Also shown in Figure 4 are the effects of constraining the top of the model to remain horizontal as it deforms, which are significant for longer edge cracks. The addition of this boundary condition is equivalent to adding a compressive load to the top of the model above the cracked region, reducing the elastic stresses and hence the Y and K values. Figure 4 also shows the polynomial fitted to these geometry correction factor values.

**Figure 5:**
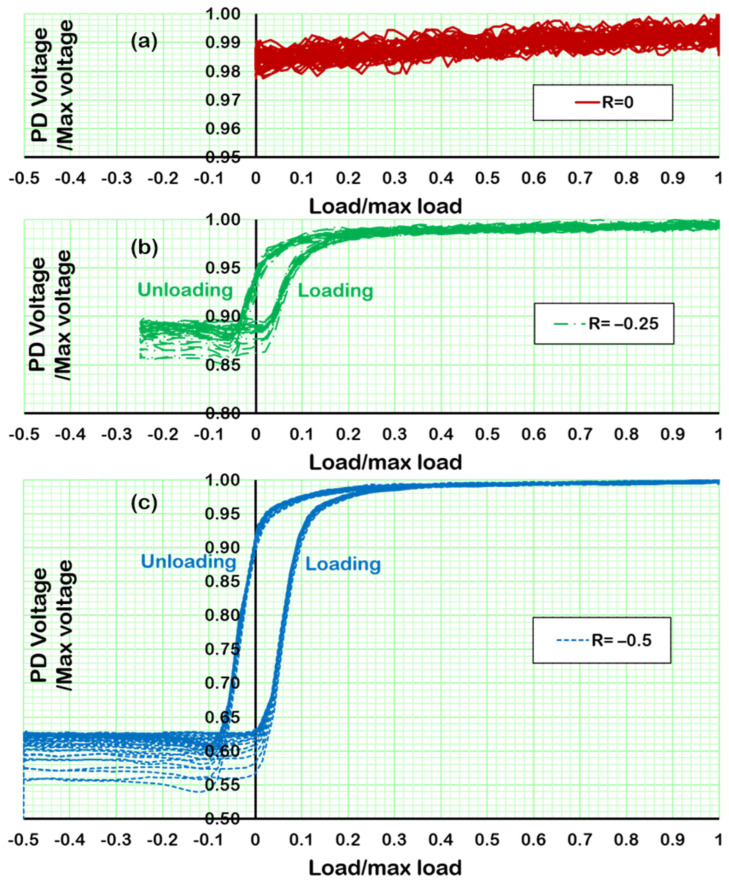
Hysteresis loops of PD voltage against applied load for (**a**) R = 0, (**b**) R = −0.25 and (**c**) R = −0.5, FG RR1000 material at 20 °C.

The contact arrangement shown in Figure 2 does not currently account for plasticity in the crack wake, which could cause the crack to close at a higher load level at R = 0 loading conditions than predicted by the model and affect the calculated crack tip stress and strain ranges. To understand whether this was an issue, measurements of Potential Drop (PD) voltage were taken throughout twenty loading cycles at R-ratios of 0, −0.25 and −0.5 using FG RR1000 corner crack test pieces as shown in Figure 5. As shown in Figure 5a, the plot indicates negligible closure at R = 0, and hence it is not necessary to include crack wake plasticity in the corresponding FE models. The crack closes fully at the other two R-ratios, which generates hysteresis in the load–voltage traces.

Additional sensitivity calculations included the use of a 1 µm notch radius, non-linear geometry and different values of Young’s modulus and Poisson’s ratio, all of which gave Y values within 0.7% of the datum FE results. The elastic stress–distance profile close to the notch root in the crack growth direction was also checked to confirm that it captured the expected 1/√r singularity well.

Having determined that the modelling method was appropriate to represent the crack tip stress fields, plastic R = 0 FE analyses were run at nineteen different peak loads representing K_max_ values from 1–86 MPa√m. Isotropic plasticity was used with a single cycle zero-max-zero loading history. The plasticity data for FG RR1000 at 20 °C were expressed in true stress-log strain form and was supplied by Rolls-Royce plc. Isotropic rather than kinematically hardened plasticity was used because the material close to the crack tip experiences higher absolute strain levels than it has seen previously with each load reversal. Rolls-Royce has previously conducted complex strain-controlled deformation tests with blocks of 100 loading cycles at the same conditions followed by an increase in strain amplitude and also through-zero strain-controlled tests going to a higher strain magnitude each time the loading was reversed. In both of these test types, taking the material to higher absolute levels of strain than it had previously experienced made the slope of the stress–strain response revert immediately to that of the monotonic tensile curve. Using isotropically hardened plasticity in the FE analyses reproduces this behaviour.

### 2.2. Calculation of R = 0 Crack Growth Rates Based on Fatigue Damage Accumulation Ahead of the Moving Crack Tip Stress Field

**Figure 6:**
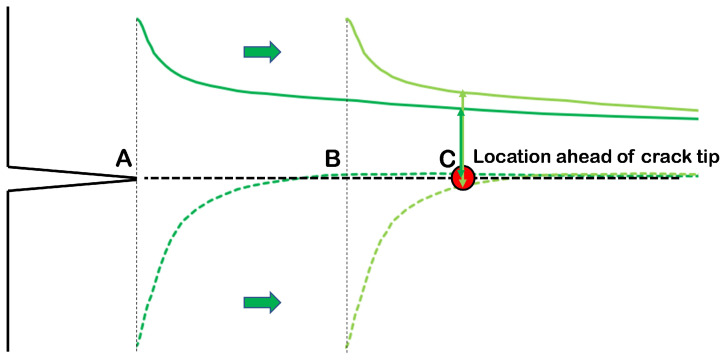
Increasing maximum and minimum stress fields experienced at a location as the crack tip moves towards it.

Figure 6 shows the general principle behind representing crack growth as progressive material failure due to the accumulation of damage from successive fatigue loading cycles as the crack tip moves towards a point in the crack path. Initially the crack tip is at position A, and the maximum and minimum plastic stress–distance profiles associated with the loading cycle extremes are shown by the darker curves. At a location C, some distance ahead of the crack tip, the amplitude of the fatigue loading cycle will be relatively modest. As the crack grows and the tip reaches B, however, the stress range experienced at location C increases, reaching a maximum value when the crack tip approaches that point.

To calculate crack growth rates, the plastic maximum and minimum stress fields for a selected K_max_ value and R-ratio are moved towards the location where the fatigue damage is calculated in 1 µm steps in an Excel spreadsheet, starting with the notch root 300 µm away. At each step, the fatigue damage in N loading cycles (damage = N/N_f_ where N_f_ is the number of cycles to failure) is calculated using a Basquin-style [22] life equation based on the Walker [23]-corrected equivalent 0-max stress at the current distance from the crack tip as shown in Equations (2) and (3). A threshold stress *σ_th_* below which no damage occurs is also included in the life equation. The fatigue damage values from each step are summed, and a value of N is calculated such that the total fatigue damage at the calculation location is equal to unity when the crack tip reaches it. The crack growth rate for this geometry and loading condition is then the size of the crack tip movement step (1 µm) divided by N.

(2) $$\frac{\left({∆\sigma }_{equiv\;0-max}-\sigma_{th}\right)}{UTS}=\alpha .{\left(N_{f}\right)}^{\beta }$$

(3) $${∆\sigma }_{equiv\;0-max}=∆\sigma {\left(1-R\right)}^{\left(m-1\right)}$$

In Equations (2) and (3) σ_th_ is the threshold value of equivalent 0-max stress, UTS is the material’s Ultimate Tensile Strength, a and b are fitting parameters, and m is the Walker exponent.

The S–N (stress–life) curve used to calculate the fatigue damage ahead of the crack tip was fitted to FG RR1000 LCF test results from plain specimens run under strain and load control at two different loading R-ratios. Unfortunately, no results were available at 20 °C, and therefore data from 300 °C tests were used instead. The fatigue lives at this temperature are expected to be similar to but slightly lower than those at 20 °C for equivalent loading conditions. This temperature read-across is considered to be reasonable because

(i) the crack growth behaviour is similar at the two temperatures (see the Rolls-Royce design data later in Figure 8);
(ii) normalizing the stress range in Equation (2) by the material’s tensile strength generates slower predicted growth rates at 20 °C than 300 °C;
(iii) the method used to fit the fatigue data allows for some small adjustments to be made to the crack growth rate as will be described later.

The load controlled test lives are to failure, and the strain controlled lives are to a 10% load drop relative to stabilised stress conditions. Two different surface conditions were used for the fatigue tests, polished and shot peened, although the lives were similar, and therefore the data were combined.

The strain controlled tests were analysed using a single element FE model through one loading cycle with isotropic plasticity and 20 °C material properties to calculate maximum and minimum stresses. This is consistent with the calculation method used to determine the maximum and minimum crack tip stress fields and also helps to scale the test results to 20 °C, the temperature that will be used for the crack growth rate predictions.

**Figure 7:**
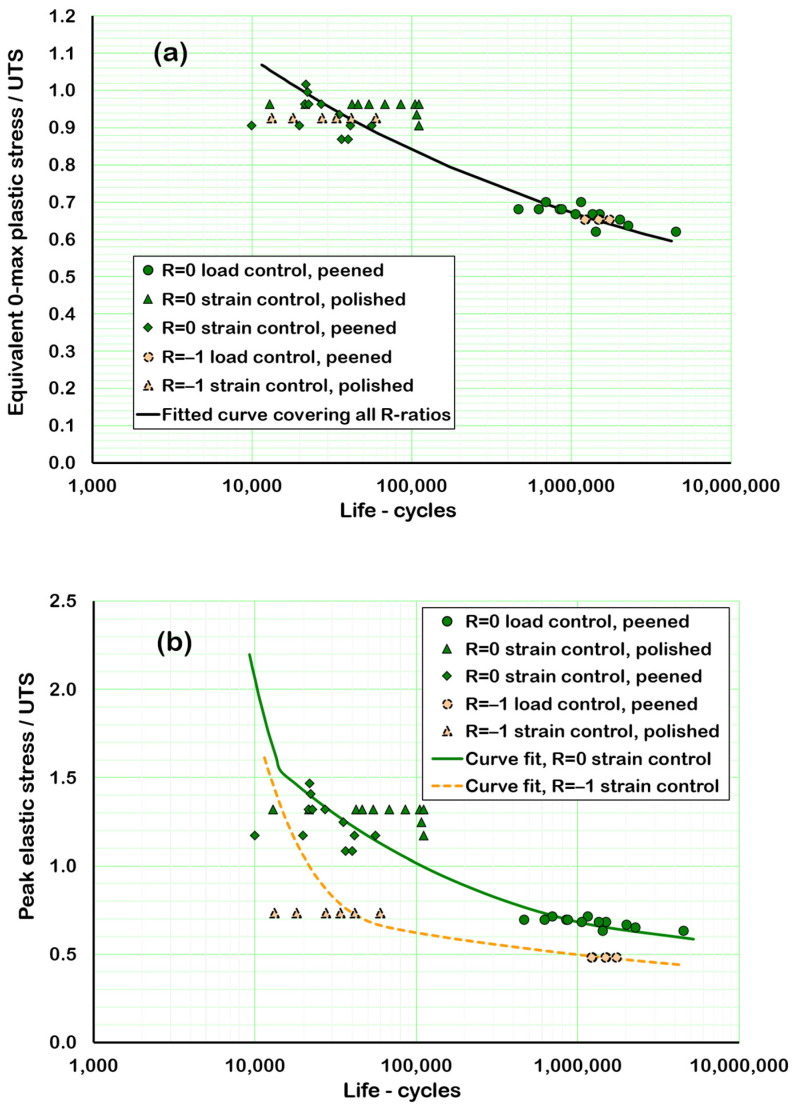
Fitted fatigue curves based on plastic stresses as a function of (**a**) equivalent 0-max plastic stress/UTS and (**b**) peak elastic stress/UTS, with fine grain RR1000 material at 300 °C.

The fitted values of the parameters m, σ_th_/UTS, a and b were 0.44, 0.31, 3.60 and −0.17. This gives the curve shown in Figure 7a, which collapses the test data well for all the different loading conditions. Because the relationship between equivalent 0-max plastic stress and peak elastic stress is different for the two R-ratios, Equations (2) and (3) generate separate curves on peak elastic stress–life axes as shown in Figure 7b. The full curve shapes shown in this plot were predicted by running additional single element plastic FE simulations at a wide range of R = 0 and R = −1 strain-controlled loading conditions.

The da/dN–ΔK curve prediction depends solely on the stress fields ahead of the crack tip from the plastic FE analyses and the fit to the plain test piece LCF data. When the crack tip is close to the calculation position, however, the stresses are very high, and the corresponding fatigue damage rate comes from extrapolating the curves in Figure 7 to low lives. It is possible for this extrapolation to be varied slightly, changing the predicted crack growth rates by up to a factor of two, without upsetting the fit to the test data. An improved fitting method was therefore developed that had inputs of both the LCF data and typical crack growth Paris law-based design data for FG RR1000 at 20 °C. The curves shown in Figure 7 use this fitting method.

**Figure 8:**
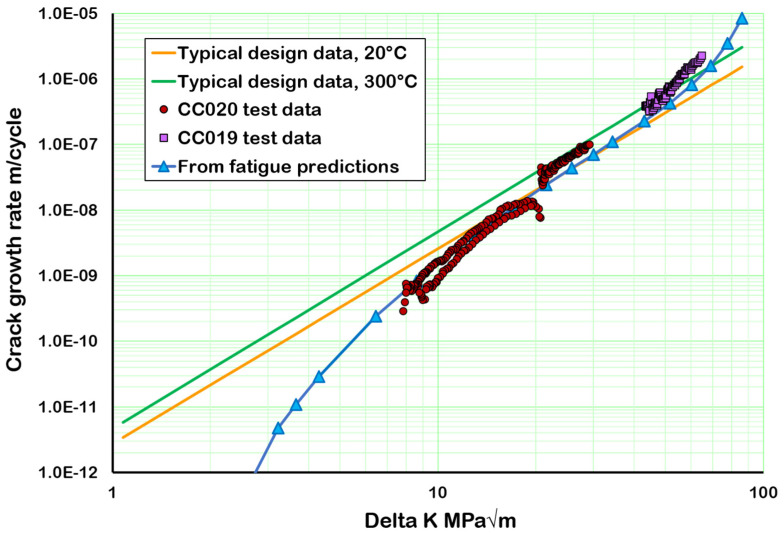
Comparison of predicted 20 °C R = 0 crack growth rates from the fatigue analogy method with experimental 20 °C data and database Paris law values at 20 and 300 °C with FG RR1000.

The resulting predicted crack growth rate curve for R = 0 is compared with typical corner crack-derived design data and test results from the current research programme in Figure 8. The curve exhibits threshold-like behaviour at low K values, and the slope also increases at high K values due to the FE model experiencing plastic strain across the whole ligament of material ahead of the crack. In the ΔK range of 15–40 MPa√m, where most experimental data are usually collected, the growth rate–ΔK relationship is approximately linear, and its slope is very close to the database value. Overall the agreement between the predictions and the test and design data is extremely good. The calculated threshold ΔK is a little low compared to the design database value of 8 MPa√m, although the predictions are consistent with the CC020 test data at low ΔK values.

**Figure 9:**
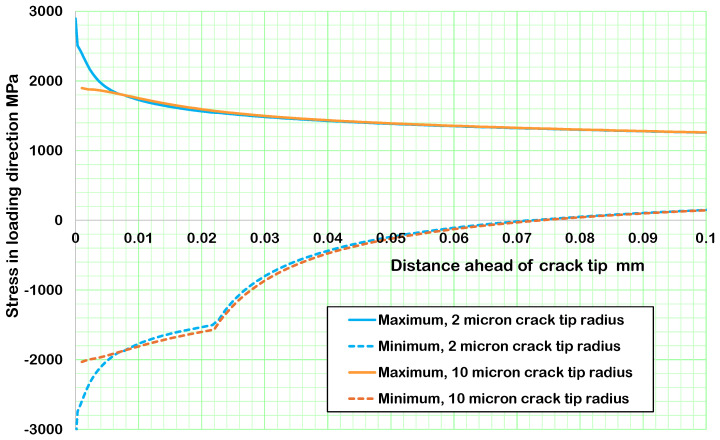
Comparison of stress–distance profiles at the maximum and minimum applied loads from analyses with crack tip radii of 2 and 10 μm, R = 0, and K_max_ = 30 MPa√m.

The sensitivity of the predictions to the size of the modelled notch was assessed by running additional FE analyses with a 2 μm radius, which was the smallest that consistently converged the plastic runs. The maximum and minimum stress–distance profiles from R = 0 analyses with a *K_max_* of 30 MPa√m are shown in Figure 9. As expected, the profiles are very similar apart from very close to the notch root. Because the areas under the two must be the same to satisfy equilibrium, the build-up of fatigue damage is actually slightly higher for the 10 μm radius until the crack tip is 5–6 μm away from the assessment location. At this condition around 0.75 of the fatigue life has been consumed, and the maximum difference in growth rates from the two FE analyses is the inverse of this number, i.e., a factor of 1.33, which does not significantly affect the conclusions about the successful performance of the model.

In addition, it is unlikely that the stress gradients predicted within 6 μm of the smaller radius crack tip can be sustained within a single grain of RR1000 material, which has a mean size of around 11 μm. When crack tip blunting is also considered, which will happen in reality but is not included in the linear geometry assumed for the FE runs, it is likely that the relatively smooth stress and strain gradients produced by the 10 μm radius notch model will be an appropriate representation of the real material behaviour.

### 2.3. Growth Rate Predictions for Other R-Ratios

**Figure 10:**
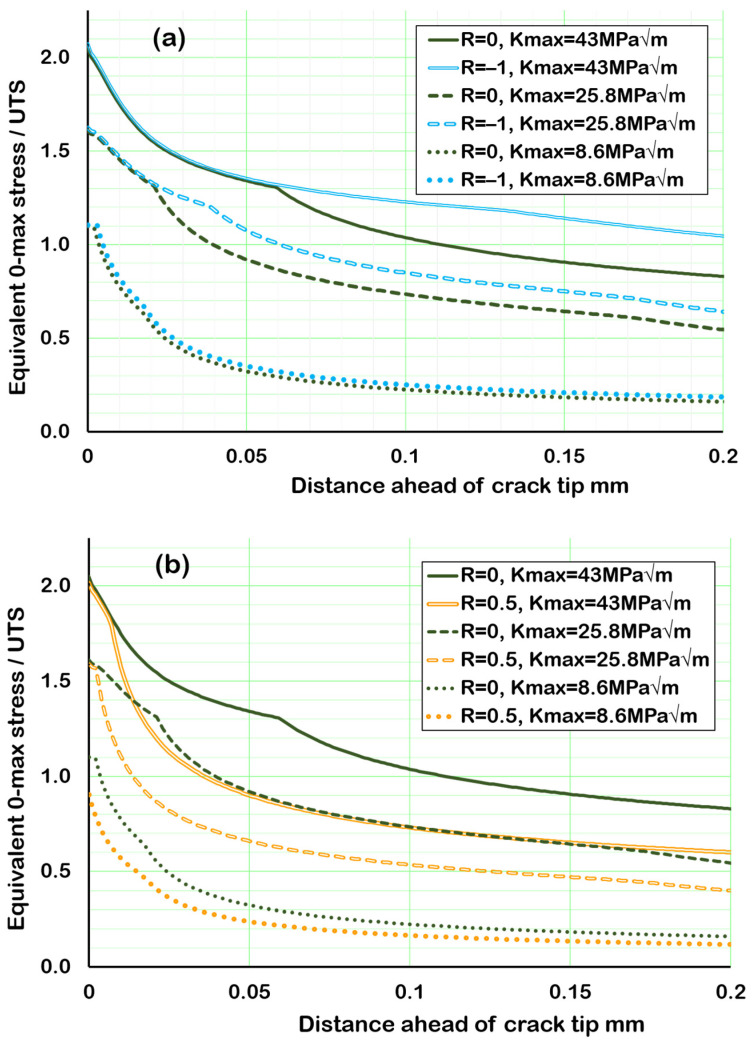
Comparison of equivalent 0-max stress–distance profiles ahead of the crack tip from (**a**) R = 0 and R = −1 loading profiles and (**b**) R = 0 and R = 0.5 loading profiles at different K_max_ values.

The effects of loading R-ratio on crack growth rates were then studied by running two further series of 2D plane stress analyses at R = −1 and R = 0.5. As shown in Figure 10a, close to the crack tip, the plastic equivalent 0-max stress–distance profiles for the same peak applied load at R = 0 and R = −1 are very similar, and therefore the predicted growth rates for R = −1 were only slightly faster despite the applied load range being twice that at R = 0 conditions. This trend was consistent across the range of applied loads and lines up extremely closely with the behaviour seen in tests conducted by Rolls-Royce.

**Figure 11:**
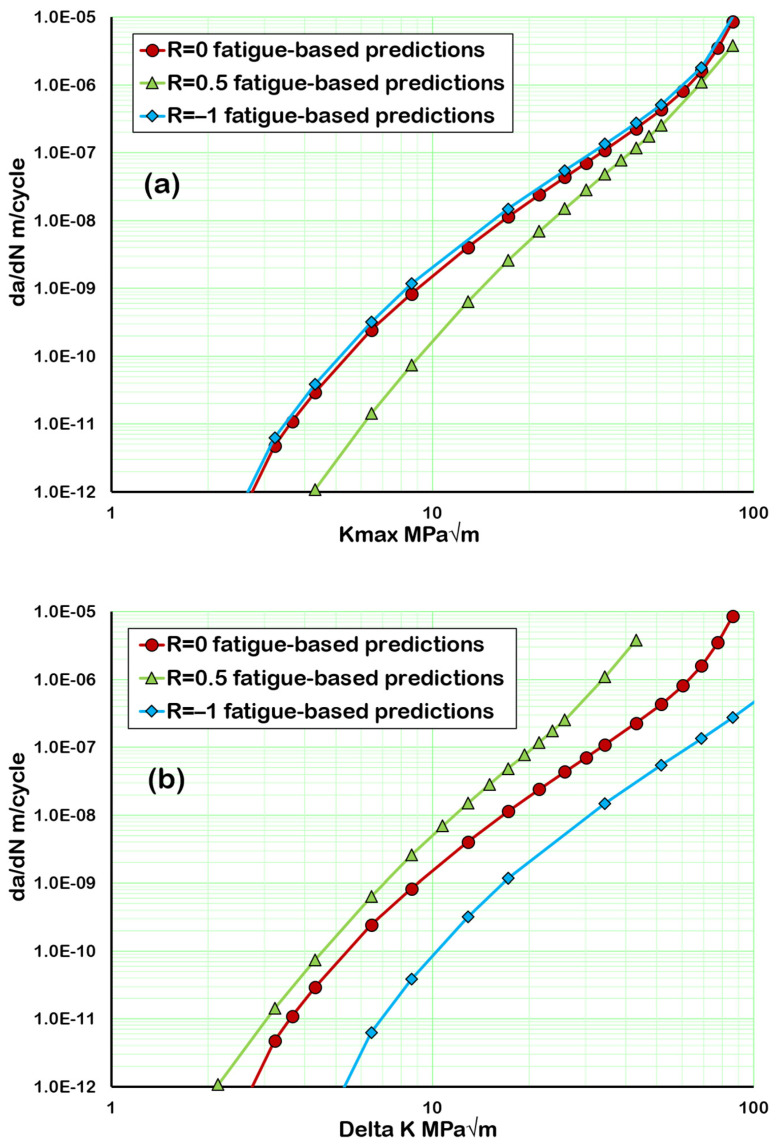
Predicted R = 0, −1 and 0.5 crack growth curves for FG RR1000 material at 20 °C based on stress–distance profiles from 2D plane stress analyses and plain test piece LCF data as a function of (**a**) K_max_ and (**b**) ΔK.

The trends are different, however, between R = 0.5 and R = 0 as shown in Figure 10b. The R = 0 equivalent stress–distance profiles are significantly higher than those for R = 0.5 at low K_max_ values. As K_max_ increases, the profiles then start to coalesce at the crack tip location and the predicted fatigue damages, and hence crack growth rates become more similar. This can be seen in Figure 11a. The interesting thing about this figure is that it clearly shows that whilst the da/dN–ΔK curves are parallel for R = 0 and R = −1, the predicted R = 0.5 curve is steeper. Data available in the literature (Dinda [24], Zheng [25], Bulloch [26] and Huang [27]) go against this prediction, however, and often suggests that the R = 0.5 crack growth curves are flatter than for R = 0.

An R = 0.5 laboratory test was therefore run at 20 °C to check out this aspect of the model’s behaviour, and a comparison was also made between the Paris line slopes fitted to fast loading cycle R = 0, R = −1 and R = 0.5 tests performed by Rolls-Royce plc, Derby, UK on FG RR1000 material, albeit at much higher temperatures.

**Figure 12:**
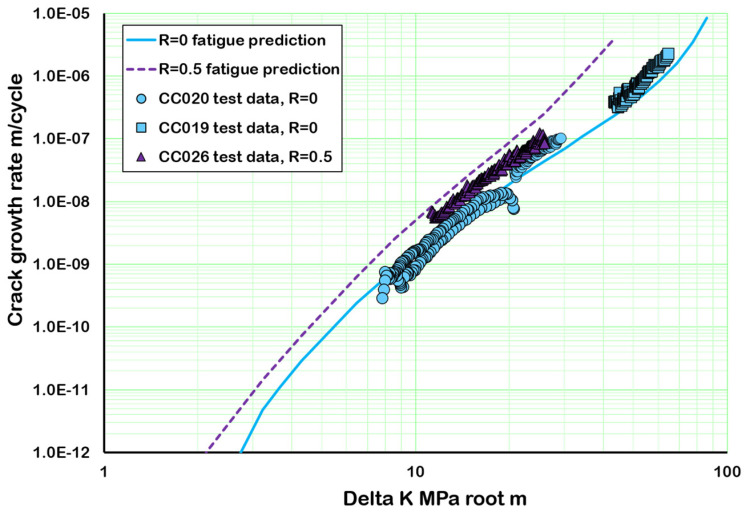
Comparison of R = 0 and R = 0.5 predictions from the fatigue analogy method with test data, FG RR1000 material at 20 °C.

Growth rates from the test are compared in Figure 12 with the R = 0 results shown previously, but there is no significant difference in slope between the two growth rate curves.

**Figure 13:**
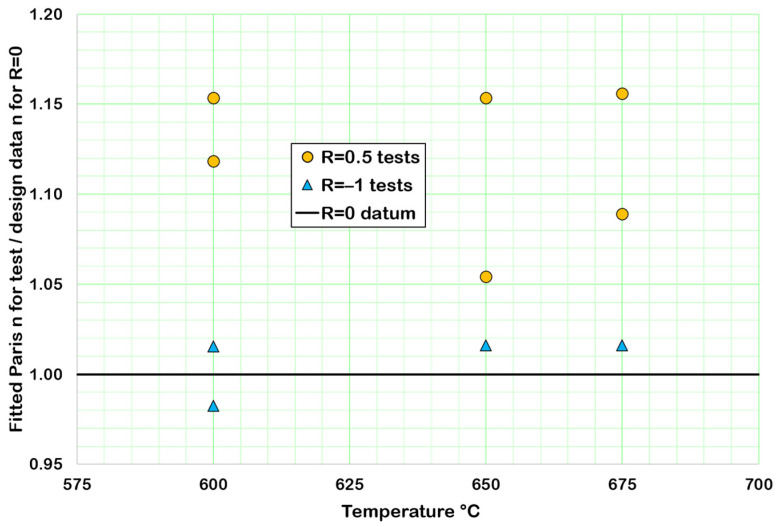
Comparison of Paris line slopes fitted to individual tests at R = 0.5 and R = −1 with design data values for R = 0: FG RR1000 material at higher temperatures.

For the higher-temperature tests shown in Figure 13, however, there do seem to be systematic trends in that the Paris slopes fitted to individual R = 0.5 tests are higher than the database values for R = 0 (shown as the solid horizontal line) and the slopes fitted to R = −1 tests are very similar to those for R = 0. These trends are consistent with those predicted by the model at 20 °C, and although the temperatures are very different there will only be a very small amount of time dependent crack growth in the hotter tests because of their high loading frequency.

### 2.4. Overload Behaviour Predictions

Finally, the expected R = 0 growth rates following an overload were predicted using the fatigue analogy. This is a much more complex calculation and was implemented in Excel using a Visual Basic macro rather than formulae in specific worksheet cells. This greatly reduced the physical size of the spreadsheet and allowed a finer spatial resolution of 0.1 µm per calculation step and an overall calculation distance of 1 mm to be used. It also allowed the K values and stress fields to be modified as the crack moves and grows. The main inputs to the macro were, as previously, plastic maximum and minimum stress–distance profiles for a wide range of K_max_ values and in addition the peak applied load as a function of crack length.

**Figure 14:**
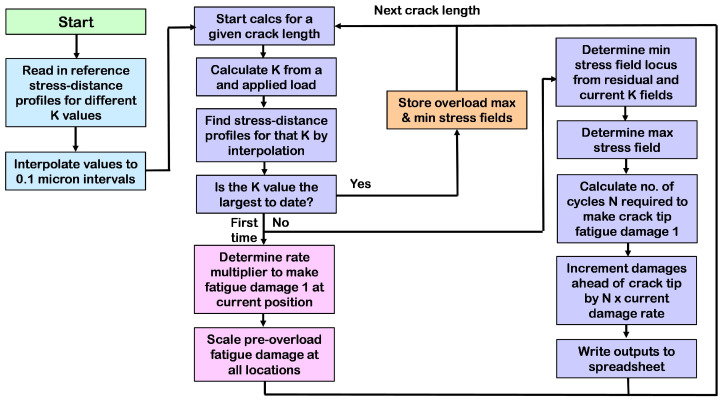
Excel macro flowchart for calculating overload behaviour using the fatigue analogy for crack growth.

The calculation process for the spreadsheet macro is shown in Figure 14. Based on the K_max_ value calculated for the starting crack length and peak datum cycle load using the edge crack geometry correction factor polynomials for constrained conditions shown in Figure 4, initial plastic stress–distance profiles are determined by interpolation between the closest sets of FE results. Increments of fatigue damage are then calculated at all the locations in the 1 mm calculation length. The crack tip position is then moved progressively through the material, and the above calculation process is repeated, with the fatigue damage increments at each location ahead of the crack tip being summed, until the change in applied load associated with the overload is found in the input data. At this time, the fatigue damage calculated during all the previous timesteps and at all locations within the material is scaled by the same factor such that the value at the current crack tip position is unity. This allows the da/dN–ΔK behaviour to date to be output.

To describe the subsequent crack growth, a number of assumptions first have to be made about how the minimum (residual) stress field associated with the overload cycle combines with the moving stress fields from the subsequent cycles at the datum load level:

- Because the residual compressive stress field resulting from the overload is generated by a localised inelastic strain distribution it is assumed to remain fixed in space and have the same stress magnitudes as the crack grows;
- When the crack tip has moved to a distance x ahead of the overload position, the peak tensile stress in the cycle at that location is given by the overload residual stress at x plus the crack tip stress range corresponding to the K value from the datum loading at the new crack length;
- As the crack grows further, it will reach a position where the overload residual stress field at x is less compressive than the minimum crack tip stress field associated with the R = 0 loading at the current K range. The minimum crack tip stress is then assumed to be that from the R = 0 analysis. This is because the maximum and minimum crack tip stress fields are assumed to equilibrate at this level: additional yielding at one extreme of the loading cycle would generate higher stresses in the opposite direction at the other end of the loading cycle which would return the stresses to their starting values;
- Ahead of the crack tip, the minimum stress is assumed to be the more compressive of that from the residual stress field and the R = 0 analysis at the current K range;
- The maximum stress field is determined by adding the stress range–distance profile for the current K value to the minimum stress field.

The implementation of these assumptions was checked in detail by comparing stress–distance profiles produced by the spreadsheet against the behaviour from an analysis where, after an overload, bands of elements ahead of the crack tip were removed progressively to grow the crack and evolve the stress fields.

As described previously, at the crack length where the overload occurs all of the calculated fatigue damages are scaled by the same factor such that the value at the crack tip is unity. The fatigue damage at the next location ahead of the crack tip will then be close to this value. The crack tip is moved to that point, and using the above assumptions first, the minimum and then the maximum stress field are determined by combining the contributions from the residuals and the current loading. The corresponding fatigue damage rates per cycle are then calculated at all the assessment locations. At the new crack tip location, the number of additional cycles required to cause the material to fail is calculated based on the difference between unity and its previous damage. The current damage rate at all the other locations ahead of the crack tip is multiplied by this number of cycles and then added to the previous fatigue damage values.

This calculation process is repeated to grow the crack, with the number of cycles required to fail the current ligament of material always varying because of the changing stress fields and different amounts of prior fatigue damage. Crack growth rates throughout this process are defined as the calculation step length divided by the number of cycles to fail each successive ligament.

The crack growth rate predictions following an overload were then compared with experimental data from a room-temperature R = 0 corner crack growth test, with overloads to twice the normal cycle amplitude at datum K_max_ values of 17.3 and 25.8 MPa√m and run at Swansea University on FG RR1000 material. Each overload was modelled separately in the spreadsheet, starting the calculations at a crack length 0.3 mm smaller than that associated with the overload and continuing for a further 0.7 mm. Note that because of the different geometry correction factors for corner and edge cracks the modelled crack lengths for the overload cycles do not match those from the test, although the K values and stress fields are the same.

**Figure 15:**
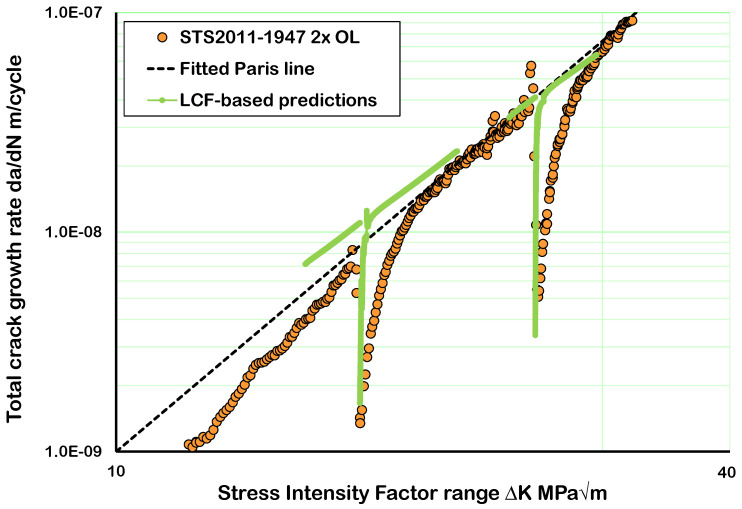
Comparison of overload crack growth rate predictions made based on fatigue behaviour with test data: R = 0 corner crack growth test with single cycle overloads to twice baseline magnitude at K_max_ values of 17.3 and 25.8 MPa√m, using fine grain RR1000 material at 20 °C.

The experimental data and the model predictions are shown in Figure 15. The dashed black Paris line was fitted to those parts of the experimental data that were unaffected by the overloads.

Following both overloads, the predicted growth rates return to the datum cycle values when the extremes of the plastic zones from the current load and the overload are coincident. This behaviour results from the rules used to generate the stress fields following the load drop and is consistent with the assumptions made in the Wheeler [28] and Willenborg [29] overload retardation models.

For both overloads, the predictions of the reduced crack growth rates immediately after the overload are very good. The general curve shapes also look reasonable and are an improvement over those generated by the Wheeler and Willenborg models, which have a discontinuity in the growth rate where they return to Paris behaviour. The duration of the overload effects is under-predicted, however, which has been seen consistently in similar tests run at Swansea University and is considered to be associated with using corner crack test pieces.

In addition, the calculations show oscillations in the predicted crack growth rates as can be seen in the simulation of the first overload. This is thought to result from using finite width calculation steps and stress fields that sometimes have fluctuations and sub-surface peaks when the residuals from the overload are included. These stress field irregularities can cause the next calculation point to accumulate almost as much fatigue damage as at the current crack tip location. Very few cycles are then required to reach failure there, resulting in a high crack growth rate. The damage one step ahead of the new crack tip position will only have increased by a small amount during this step, however, and it may then require a higher number of additional cycles before it becomes unity. This results in a slow predicted crack growth rate in this increment, and the behaviour can persist, resulting in oscillations in the predicted growth rate.

Overall, the method clearly shows promise in modelling post-overload behaviour and merits additional work to eliminate the currently predicted growth rate oscillations.

## 3. Conclusions

The finite element analysis style used has been shown to reproduce established geometry correction factors for edge cracks (and, in other work by Williams [30], for 3D corner cracks) very closely.

It has also been shown that a stress-based curve fit performed to fatigue test results on plain smooth FG RR1000 specimens can be used to generate R = 0 low-temperature crack growth rates that agree extremely well with database values. The predicted threshold ΔK value, however, is slightly lower than that obtained from crack growth tests.

Use of the calculation method to predict crack growth behaviour at other loading R-ratios has provided an analytical basis for the similarity in growth rates observed in R = 0 and R = −1 loading cycles at the same peak load. The prediction of a slightly increased da/dN–ΔK curve slope at R = 0.5 was unexpected but was shown to be consistent with data generated on the same material at higher temperatures.

Good predictions of post-overload behaviour were also made using the fatigue analogy for crack growth. The current calculation method would benefit from improvements, however, to reduce the oscillations that are sometimes seen in the predicted crack growth rates.

Overall, the calculation framework presented in this paper for relating low cycle fatigue and crack growth data has shown significant promise and warrants further development.

## Data Availability

The original contributions presented in the study are included in the article, further inquiries can be directed to the corresponding author.

## Abbreviations

The following abbreviations are used in this manuscript:

a	Crack length
α	Lifing curve parameter related to its intercept
β	Lifing curve parameter related to its slope
ΔK_eff_	Effective stress intensity factor range
K	Stress intensity factor
K_max_	Maximum stress intensity factor in loading cycle
K_op_	Stress intensity factor at which the crack is fully open
m	Walker exponent
N	Number of applied fatigue cycles
N_f_	Number of fatigue cycles to failure
PD	Potential drop
r	Distance ahead of the crack tip
ρ*	Notch root radius and calculation block size in the Noroozi model
σ_th_	Threshold stress value in fatigue curve equation
Y	Geometry correction factor in stress intensity formula
